# Validation and validity of verbal autopsy procedures

**DOI:** 10.1186/1478-7954-9-22

**Published:** 2011-08-01

**Authors:** Daniel Chandramohan

**Affiliations:** 1 London School of Hygiene and Tropical Medicine, Keppel Street, London WC1E 7HT, UK

## Commentary

Methods for interpreting verbal autopsy (VA) that have been validated fall into two major categories: (1) physician-certified verbal autopsy (PCVA), the commonly-used method in which one or more physicians ascertain causes of death based on their clinical judgment; and (2) computerized coding of verbal autopsy (CCVA), in which causes of death are derived using predefined criteria. Decision rules for CCVA can be expert opinion-based or data driven. The accuracy of these VA interpretation methods varies depending on causes of death per se, while the effect of misclassification error in VA on the estimates of cause-specific mortality fractions (CSMF) depends on the distribution of causes of death. The importance of acknowledging the effects of misclassification of causes of death by VA has been highlighted by the recent controversial estimates of malaria mortality in India [1]. The parameters of validity of VA obtained from a validation study may be useful to measure the uncertainty limits of CSMFs due to misclassification errors of VA, and in some contexts, to adjust the estimate of CSMF for the effect of misclassification error [2,3].

The gold standard diagnosis of cause of death (COD) for assessing the validity of VA has been the COD derived from hospital medical records. The main limitations of using hospital-based CODs as the gold standard are: (1) The accuracy of medical records-based COD is debatable, even though some studies have refined the diagnosis with expert review of hospital records; and (2) the composition and distribution of hospital CODs may not be representative of deaths occurring in the community. In addition, if diagnostic algorithms for CCVA are developed from subsets of validation study datasets, their external validity may be compromised. Nevertheless, hospital diagnosis of COD based on defined clinical and laboratory criteria are the only useful gold standard available at present for validating VAs.

The validity of InterVA has not previously been tested against a gold standard diagnosis. The reliability of InterVA has been determined by examining the concordance of CSMFs estimated by InterVA and PCVA. Given that the accuracy of PCVA is questionable, estimating concordance between causes of death derived by PCVA and InterVA as a measure of validity needs to be interpreted with caution.

Measures used to assess the validity of VA include sensitivity, specificity, positive predictive value, and absolute (absolute error) or relative (relative error) difference between CSMF estimated by VA and true CSMF in the validation data. Sensitivity and specificity that measure accuracy at the individual level vary substantially between causes of death across different VA interpretation methods. The absolute and relative errors of CSMF measure the accuracy of VA at the population level. The variability of the absolute error in CSMF appears to be reasonable for most CODs because often the number of false positive and false negative diagnoses balance out. However, the relative error in CSMF tends to be exaggerated, especially if the CSMF is low.

Murray and colleagues in this series recommend determining the validity of VAs using cause-specific and average chance-corrected concordance across causes for single cause assignment methods, as well as for one to k causes across causes for individual multiple cause assignment methods [4]. For estimation of CSMFs, they recommend CSMF accuracy and cause-specific concordance correlation coefficients of estimated CSMFs compared to true CSMFs. These measures are useful to compare the performance of different VA interpretation methods and could also be used to estimate the uncertainty limits of CSMF estimates attributable to misclassification errors of VA. Methods to estimate uncertainty limits for CSMFs attributable to misclassification errors of VA need to be further developed.

Flaxman et al [5] have developed and validated a new CCVA, the Random Forest (RF) Method, for interpreting VA in a large multicountry validation dataset. The median chance corrected concordance rate of the RF Method is higher than PCVA for adult, child, and neonatal VAs. These are very promising results and if confirmed in other validation datasets, software for coding VAs based on the RF Method would greatly improve the reliability and timeliness of CSMFs collected using VAs. What is urgently required is an objective assessment of the performance of the RF Method versus InterVA, based on this high-standard VA validation study dataset, and then to actively promote and facilitate the implementation of the best-performing method in all mortality surveillance systems using VAs. This would likely greatly improve the quality and comparability of cause-specific mortality data obtained using VAs.
